# Testing a linear time invariant model for skin conductance responses by intraneural recording and stimulation

**DOI:** 10.1111/psyp.12986

**Published:** 2017-09-01

**Authors:** Samuel Gerster, Barbara Namer, Mikael Elam, Dominik R. Bach

**Affiliations:** ^1^ Division of Clinical Psychiatry Research University of Zurich Zurich Switzerland; ^2^ Neuroscience Centre Zurich University of Zurich Zurich Switzerland; ^3^ Department of Physiology and Pathophysiology University of Erlangen Erlangen Germany; ^4^ Department of Clinical Neurophysiology The Sahlgrenska Academy of Gothenburg University Gothenburg Sweden; ^5^ Wellcome Trust Centre for Neuroimaging University College London London United Kingdom; ^6^ Department of Psychiatry, Psychotherapy, and Psychosomatics University of Zurich Zurich Switzerland

**Keywords:** microneurography, psychophysiological model, skin conductance, sudomotor nerve, sympathetic nervous system

## Abstract

Skin conductance responses (SCR) are increasingly analyzed with model‐based approaches that assume a linear and time‐invariant (LTI) mapping from sudomotor nerve (SN) activity to observed SCR. These LTI assumptions have previously been validated indirectly, by quantifying how much variance in SCR elicited by sensory stimulation is explained under an LTI model. This approach, however, collapses sources of variability in the nervous and effector organ systems. Here, we directly focus on the SN/SCR mapping by harnessing two invasive methods. In an intraneural recording experiment, we simultaneously track SN activity and SCR. This allows assessing the SN/SCR relationship but possibly suffers from interfering activity of non‐SN sympathetic fibers. In an intraneural stimulation experiment under regional anesthesia, such influences are removed. In this stimulation experiment, about 95% of SCR variance is explained under LTI assumptions when stimulation frequency is below 0.6 Hz. At higher frequencies, nonlinearities occur. In the intraneural recording experiment, explained SCR variance is lower, possibly indicating interference from non‐SN fibers, but higher than in our previous indirect tests. We conclude that LTI systems may not only be a useful approximation but in fact a rather accurate description of biophysical reality in the SN/SCR system, under conditions of low baseline activity and sporadic external stimuli. Intraneural stimulation under regional anesthesia is the most sensitive method to address this question.

## 1 INTRODUCTION

Skin conductance responses (SCR) are often measured to make statements about psychological processes such as cognitive load, emotional arousal, threat prediction, or motor preparation (Boucsein, 2012). Such inference is traditionally embodied in operational methods by which some data features are taken as indicators of the psychological process. These methods imply models of how SCR are generated (Bach & Friston, 2013). Model‐based analysis makes such implicit models transparent and explicit in mathematical form (Alexander et al., 2005; Bach, Flandin, Friston, & Dolan, 2009, 2010; Benedek & Kaernbach, 2010a, 2010b; Greco, Valenza, Lanata, Scilingo, & Citi, 2015; Lim et al., 1997). This allows estimating parameters of the assumed psychological process from measured data. Probabilistic estimation of psychological states, as, for example, embodied in the Psychophysiological Modelling (PsPM) framework, can furnish higher signal‐to‐noise ratio than operational methods (Bach, 2014; Bach, Daunizeau, Friston, & Dolan, 2010; Bach, Daunizeau, Kuelzow, Friston, & Dolan, 2011; Bach et al., 2009; Bach, Friston, & Dolan, 2010, 2013; Bach & Staib, 2015; Staib, Castegnetti, & Bach, 2015). Such enhanced sensitivity motivates further development of this approach.

Since Alexander et al.'s (2005) work, all published models have split the relation between psychological process and SCR into two systems. The first is a neural system that transforms a psychological process into firing bursts of the peripheral sudomotor nerve (SN). The second is a peripheral (effector organ) system that translates SN bursts into actual SCR and summarizes the activity of SN end terminals, neurotransmitter diffusion, and the operation of sweat glands (Boucsein, 2012). While assumptions about the neural process are heterogeneous, all approaches converge in modeling the SN/SCR mapping as a linear time‐invariant (LTI) system. This is a system with two defining properties: first, the output does not explicitly depend on time (time invariance), and second, the response to several inputs is the sum of the responses to the individual inputs (linearity). An LTI system is unambiguously specified by its impulse response function (RF), that is, the output to a very brief input. If the system is fully known, biophysical relations can be exploited to analytically derive a RF (e.g., for fMRI: Buxton, Wong, & Frank, 1998). Such RF has also been proposed for SCR (Alexander et al., 2005; Benedek & Kaernbach, 2010a, 2010b; Greco et al., 2015). However, a paucity of knowledge on sweat gland biophysics may imply that these RF do not accurately reflect actual SCR. To mitigate this concern, an alternative approach is to construct a phenomenological RF on a large database of recorded SCR (Bach et al., 2009; Bach, Flandin et al., 2010; Bach, Friston, & Dolan, 2010). Such approach has also been harnessed successfully for modeling cardiac (Castegnetti et al., 2016; Paulus, Castegnetti, & Bach, 2016), pupil (Korn & Bach, 2016; Korn, Staib, Tzovara, Castegnetti, & Bach, 2017), respiratory (Bach et al., 2016; Castegnetti, Tzovara, Staib, Gerster, & Bach, 2017), and startle eyeblink responses (Khemka, Tzovara, Gerster, Quednow, & Bach, 2017).

Regardless of the RF specification, such models can only be successfully applied if the basic LTI formalism constitutes a reasonable approximation to biophysical reality. The fact that statistical sensitivity of model‐based approaches converges with, or overtakes, operational approaches (which do not make such strict assumptions) can be taken to tentatively suggest that LTI assumptions are valid for SCR. However, in the past, the validity of linear models for SN/SCR relationship has sometimes been questioned, including even the informal and loose models used in operational analysis. This criticism was mainly on the observation that the relation between SN and SCR amplitude can be variable (Bini, Hagbarth, Hynninen, & Wallin, 1980), and that repeated SN stimulation can lead to SCR with different shapes (Kirno, Kunimoto, Lundin, Elam, & Wallin, 1991; Kunimoto, Kirno, Elam, Karlsson, & Wallin, 1992a, 1992b; Kunimoto, Kirno, Elam, & Wallin, 1991). Yet, such LTI violations have so far not been quantified, and it is therefore not known to what extent and under what circumstances they would hamper the application of LTI models.

Our previous indirect tests of LTI assumptions have built on an additional neural assumption, namely, that brief sensory events cause brief SN firing with a constant latency, as suggested by direct SN recordings (Nishiyama, Sugenoya, Matsumoto, Iwase, & Mano, 2001). Thus, we have measured the linearity and time invariance of SCR to brief sensory events (Bach, Flandin et al., 2010). However, this approach cannot distinguish LTI violations in the effector organ system and deviations from the assumptions about the neural system, and thus provides only an upper bound on effector organ LTI violations. This motivates the present study in which we capitalize on two invasive methods to directly assess the effector organ, and quantify the extent of LTI violations.

The first assessment relies on simultaneous intraneural and SCR recordings (Vallbo, Hagbarth, Torebjork, & Wallin, 1979). This approach directly assesses the SN/SCR relationship. However, it is challenging to isolate the activity of SN fibers from neighboring ones with a different bursting profile (e.g., vasomotor, piloerector, or lipomotor; Macefield, Elam, & Wallin, 2002; Macefield & Wallin, 1996). Hence, intraneural recording techniques will again overestimate LTI violations, but they may usefully complement the indirect approach outlined above.

A second possibility of exploring the effector organ system's properties is engendered by intraneural SN stimulation under elimination of spontaneous peripheral nerve activity. This can be achieved by stimulating brachial nerves distal to brachial plexus anesthesia (Wallin & Elam, 1997).

In sum, the two invasive together with previously used noninvasive methods of testing LTI assumptions are influenced by different sources of noise. Hence, they may provide a convergent estimation of the extent to which LTI assumptions are fulfilled. In the present study, we combined the intraneural recording and intraneural stimulation approaches to quantitatively assess the variance in SCR that is explained in an LTI model of the SN/SCR relationship.

## 2 METHOD

### 2.1 Experiment 1: Intraneural recordings

#### 2.1.1 Participants and design

We simultaneously recorded SN activity and SCR elicited by aversive sounds, and by deviant sounds in an oddball task. Seven healthy and unmedicated volunteers (4 male, 3 female, mean age ± *SD*: 23.7 ± 4.0 years, range 19–29) were recruited from the general population and received monetary compensation for their participation. All participants gave written informed consent, and the study was approved by the local ethics committee.

#### 2.1.2 Stimulation

For each participant, 20 aversive broadband white noise sounds of 1 s length (10 ms onset and offset ramp, 95 dB sound pressure level) were delivered via headphones. For one participant, the procedure was repeated three times with breaks in between, such that this participant received overall 60 sounds. Events were separated by at least 30 s in order to unambiguously define SCR tails. Participants were tasked to press a key on a computer keyboard whenever they heard a sound.

Next, pure sinusoidal sounds with duration of 50 ms were delivered via headphones once per second, and participants were instructed to press a key when they heard a deviant sound. Standards were pitched at 440 Hz and oddballs at 660 Hz. Ten oddballs were played per experiment and constituted our events of interest. There were at least 30 standards after any oddball; the 31st–40th sounds after any oddball were equally likely to be the next oddball. The 35 first and 35 last sounds were standards.

Auditory stimulation was delivered via headphones (HD 518, Sennheiser Electronic GmbH & Co., Wedemark, Germany). All experiments were programmed in Cogent (Version 2000v1.25, www.vislab.ucl.ac.uk/Cogent) on MATLAB (Version 6.5; MathWorks; Natick, MA), and run on a personal computer with a Pentium 4 processor and a SoundMax soundcard (Analog Devices, Norwood, MA).

#### 2.1.3 Skin conductance recording

Skin conductance was recorded on the dorsal region of the right foot using 8‐mm Ag/AgCl cup electrodes (EL258, Biopac Systems Inc., Goleta, CA) and using 0.5% NaCl electrode paste (GEL101; Biopac Systems Inc.; Hygge & Hugdahl, 1985). This region is not standard for SCR recordings but allowed easy access to the efferent C fibers. The signal was recorded using a Coulbourn Lablink V system with an isolated skin conductance coupler (V15‐17 and V71‐23, Coulbourn Instruments, Allentown, PA). The output of the coupler was digitized with a sampling rate of 100 Hz (Micro1401, Cambridge Electronic Design, Cambridge, UK) and recorded (Spike2, Cambridge Electronic Design). Temperature and relative humidity of the experimental room were between 24.2–27°C and 35–48.3%.

#### 2.1.4 Intraneural recording

Standard techniques were employed to record from C fibers in the peroneal nerve (Vallbo et al., 1979). We targeted the superficial branch of the common peroneal nerve proximal to the ankle. When the tip of the recording electrode had reached a stable position in a cutaneous nerve fascicle, the skin field innervated by this fascicle was mapped by gently stroking the skin and listening to the high‐pitched sound from multifiber discharges in low threshold mechanosensitive A fibers. Neural responses were digitized with a sampling rate of 10 kHz (Micro 1401) and recorded (Spike2).

Stimulus onset was signaled by TTL pulses via the stimulus computer's parallel port and recorded together with the other data.

#### 2.1.5 Data preprocessing

Data analysis was carried out in MATLAB 8.6 (MathWorks) using PsPM 3.1 routines (pspm.sourceforge.net) and custom code that is available from the authors.

Skin conductance data were filtered with 1st order high‐pass (0.0159 Hz, corresponding to a time constant of 10 s) and low‐pass (5 Hz) Butterworth filters, *z*‐transformed to account for intersubject variability, and downsampled to 10 Hz, in line with literature recommendations (Boucsein, 2012; Boucsein et al., 2012) and previous work (Bach, Flandin et al., 2010).

**Figure 1:**
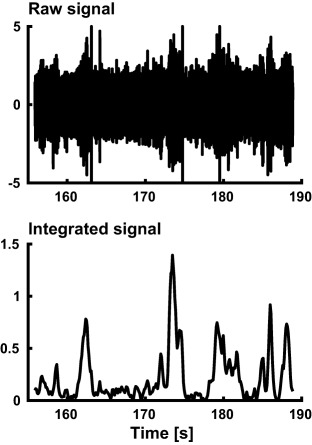
Example of an intraneural recording. The integrated nerve signal reflects the amount of multiunit activity

Intraneural recordings were preprocessed similarly to previous studies (Vallbo et al., 1979). First, we filtered with a 398th order equiripple direct‐form FIR band‐pass filter (300–4000 Hz) and *z*‐transformed. Then, a leaky integrator with a time constant of 100 ms was applied for burst detection in neuronal activity. The integrated signal was downsampled to 10 Hz and linearly detrended on 5‐s intervals to remove drift in the baseline (Figure 1).

We constructed data epochs containing the 5 s preceding and 25 s following each event onset. Data from one subject was excluded due to strong noise in SN signal. Eight individual epochs from the remaining participants were excluded due to poor SN signal quality (i.e., clipping). To derive canonical responses, we averaged the data across all epochs of the same event type from all participants, or across all epochs of the same event type from each individual participant. In contrast to previous work (Bach et al., 2009; Bach, Flandin et al., 2010), we used averages rather than principal components to account for a lower signal‐to‐noise ratio.

### 2.2 Experiment 2: Intraneural stimulation

#### 2.2.1 Participants and design

We reanalyzed data from previously published experiments (Kirno et al., 1991; Kunimoto et al., 1991) in which SCR were elicited by intraneural SN stimulation. Four healthy and unmedicated female volunteers (age range 24–49 years) were recruited from the general population and received monetary compensation for their participation. All participants gave written informed consent, and the study was approved by the local ethics committee.

#### 2.2.2 Intraneural stimulation

Stimulation was performed as described previously (Kirno et al., 1991). In short, a plastic cannula for injection of a local anesthetic was inserted into the left axilla and placed within the sheath surrounding the axillary artery and nerves. A tungsten microelectrode was then inserted into the median nerve 2–3 cm proximal to the wrist, and a similar reference electrode was placed subcutaneously a few centimeters away. A nerve fascicle innervating the index or the middle finger was impaled. Axillary nerve blockade was induced by injection of 30–35 mL of mepivacaine into the axillary cannula in order to completely inhibit spontaneous or evoked SCR. The peripheral median nerve was then stimulated at various frequencies. We analyzed stimulation frequencies between 0.1 Hz and 10 Hz.

#### 2.2.3 Recording

SCR was measured with Ag/AgCl electrodes from the area innervated by the stimulated fascicle with a Van Gogh galvanic skin reflex module that used a rise time constant of 0.3 s and a decay time of 3 s to record the first derivative of the skin conductance (Lidberg & Wallin, 1981). The signal was high‐pass filtered with 0.7 Hz cutoff.

#### 2.2.4 Data preprocessing

Data analysis was carried out using PsPM 3.1 in MATLAB 8.6 and custom code available from the authors.

The signal was first integrated to approximate the skin conductance response, by subtracting the mean and cumulatively summing the discretized signal. The reconstructed SCR signal was filtered with first‐order band‐pass (0.0159 Hz–5 Hz) Butterworth filter, z‐transformed, and downsampled to 10 Hz sampling rate.

SN stimulation rate (SR) was variable over time. We decomposed the SCR signal into 16 epochs according to stimulation rate. One epoch was excluded due to electrode malfunction. This resulted in 6 epochs with ∼0.1 Hz stimulation, 3 epochs with ∼0.2 Hz, 1 epoch with ∼0.5 Hz, 1 epoch with ∼1 Hz, 2 epochs with ∼1.5 Hz, and 2 epochs with ∼10 Hz. These 10 Hz epochs were not analyzed as this stimulation frequency exceeds by far the rate of SN bursts under physiological conditions. Each epoch contained on average 39 (10–58) events at ∼0.1 Hz stimulation, 77 (68–93) events at 0.19–0.6 Hz stimulation, and 180 (56–267) events at higher stimulation rates.

### 2.3 Modeling

Observed responses were approximated with analytic functions. SN was fitted with a Gaussian function:

(1) $$SN\left(t\right)\;=\;u\left(t\right)\;=\;\frac{A}{\sqrt{2\pi }\;\sigma }\;e^{-\frac{{\left(t-\mu \right)}^{2}}{2\sigma^{2}}}\;+\;c.$$

To estimate the parameters of this function, we used ordinary least squares (OLS) minimization and a Nelder‐Mead search algorithm as implemented in the MATLAB function fminsearch. SCR was fitted with a canonical skin conductance response function (SCRF) embodied in a third‐order constant‐coefficient inhomogeneous linear ordinary differential equation (ODE), in line with our previous approach (Bach, Daunizeau et al., 2010; Bach et al., 2011; Staib et al., 2015):

$$SCR\left(t\right)\;=\;x$$

(2) $$\dddot{x}\;+\;\vartheta_{1}\ddot{x}\;+\;\vartheta_{2}\dot{x}\;+\;\vartheta_{3}x\;+\;u\left(t\;-\;\vartheta_{4}\right)\;=\;0.$$

Parameters of this equation were estimated by free energy minimization in a variational Bayes algorithm as implemented in the toolbox VBA (Daunizeau, Adam, & Rigoux, 2014) and included in PsPM.

For Experiment 1, we averaged epochs with events of the same type, and minimum‐corrected the SCR average to account for skin conductance level that may be maintained without SN firing. We estimated response function parameters on these averages, by using the averaged SN signal as input to the ODE. This procedure was peformed either on the average from all participants, to quantify variance explained by a canonical RF, or on averages from individual participants, to quantify additional variance explained under an LTI model but with a subject‐specific RF. Notably, for some participants and event types, the resulting RF explained less variance in the individual epochs than the canonical RF. For these subjects, we used the variance explained with the canonical RF as a conservative estimate of the maximum variance that can be explained under LTI assumptions.

Next, we computed how much variance in the (epochwise minimum‐corrected) SCR data could be explained under an LTI model. We convolved the SN activity for each individual epoch with the SCRF. Because the gain factor of the SCRF is unknown, it was estimated using OLS linear regression. We computed the regression either across all epochs for each participant (assuming a fixed gain factor), or on a epoch‐by‐epoch basis (assuming a variable gain factor), and then quantified explained variance. Finally, we analyzed the estimated response gain per epoch in a linear mixed effects model (package lme4 in R) containing terms for event type (aversive stimulation, oddball), repetition (1–3 for one participant with 60 events, and 1 for the others), and epoch, as well as a random intercept. We first modeled epoch as an omnibus effect (i.e., numerator degrees of freedom equal the number of epochs per repetition, minus one), and in an exploratory analysis, more specifically we investigated a linear effect of epoch (i.e., *df* = 1).

In Experiment 2, we analyzed data epochs at particular stimulation frequencies. While in Experiment 1 there was one event per epoch, here the data contained responses to multiple stimulations per epoch that would therefore overlap, making average responses difficult to interpret. Therefore, SCRF parameters and response gain were simultaneously estimated using the VBA algorithm. We analyzed each epoch individually, all epochs for each participant, or all epochs from all participants. We modeled elicited SN activity (i.e., input into the ODE) as a series of Gaussian functions, centered on the (square wave) stimulations and with a standard deviation of 0.3 s. This differentiable approximation of the sudomotor input was chosen to facilitate the estimation; note that the truly elicited sudomotor burst will not be a square wave either. This procedure directly yielded the explained variance in the signal. Time changes in estimated response gain were analyzed in a linear mixed model as described above.

## 3 RESULTS

### 3.1 Experiment 1: Intraneural recordings

**Figure 2:**
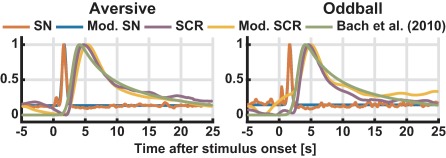
Experiment 1. Stimulus‐evoked SN and SCR, averaged over all participants and trials, together with the modeled SN/SCR (Mod. SN, Mod. SCR) and a previous SCR model (Bach, Flandin et al., 2010). To account for the previously determined average delay between foot recording (this study) and hand recording (previous model), the latter response function was shifted in time by 1.3 s (Bach, Flandin et al., 2010)

Extracted SN and SCR were averaged over all participants for each experiment and are shown in Figure 2. Averaged event‐related SCR from both experiments closely resembled results from a previous study using a larger data set (Bach, Flandin et al., 2010). We analytically approximated the average SCR, and the approximation also resembled the one from previous work (Figure 2, parameters in Table 1). This suggests that SCR elicited on the dorsal foot in the current invasive study are comparable to those elicited in more common psychophysiological experiments with standard thenar/hypothenar recordings.

**Table 1:** Model parameters from Experiment 1

ODE parameters for SCR			Gaussian parameters for SN		
Parameter^a^	Aversive	Oddball	Parameter^b^	Aversive	Oddball
ϑ_1_	1.3277	1.5457	µ	1.6850	1.6785
ϑ_2_	1.1205	1.9595	σ	0.3051	0.2471
ϑ_3_	0.1084	0.1336	A	0.7814	0.6589
ϑ_4_	−0.4265	0.3001	c	0.1567	0.0000

a ϑ1–ϑ_3_ determine the shape of the SCRF. ϑ_4_ determines the delay with respect to an eliciting SN burst and depends on the precise distance of the intraneural recording electrode from the skin; hence, it has no generalizable interpretation.

b σ describes the shape (dispersion) and µ the delay of an SN burst with respect to an eliciting external stimulus. Delay depends on recording location along the nerve. A is the amplitude, which depends on recording settings, and c is the baseline SN activity.

Averaged SN activity was modeled by a Gaussian function (Figure 2, parameters in Table 1). Standard deviation of the Gaussian was, for the two event types, 0.31 s and 0.25 s. On an epoch‐by‐epoch level, this Gaussian model explained 77.35% (aversive sounds) or 66.14% (oddball events) of SN variance when built across all participants, and 78.88% (aversive sounds) or 79.49% (oddball events) when optimizing the model per participant.

**Figure 3:**
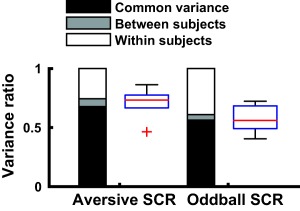
Experiment 1. Ratio of variance in SCR signal, for an aversive sound stimulation and an oddball stimulation. Bar charts show variance components under the assumption of a variable gain factor. Black: Common variance, explained by a canonical SCRF across all participants. Gray: Between‐subjects variance, additionally explained when using one SCRF per participant. White: Within‐subject variance, unexplained by the LTI models. Box plots: Variance explained with one SCRF per participant, for each participant

Next, we asked how much of the variance in the SCR signal could be explained by an LTI system that takes SN activity as input and that is described by the modeled SCRF. To do so, we convolved the epochwise SN with the SCRF, and compared it to the epochwise measured SCR (Figure 3). Because the interevent interval is long and there is thus (approximately) no summation of responses, this primarily addresses the question of time invariance. We first estimated the response gain for each epoch separately. Using a canonical SCRF across all participants, our model explained 67.84% (aversive sounds) and 56.30% (oddball events) of SCR variance. With an individual SCRF optimized per participant, the model explained 74.40% (aversive sounds) and 60.97% (oddball events) of the variance. When assuming a constant response gain across all events, explained variance was dramatically smaller (canonical/individual SCRF for aversive sounds: 39.48%/45.75%, for oddball events 34.04%/37.30%). Electrode drift (away from the nerve) and saturating sweat gland system would predict an increase or decrease of the response gain, respectively, over time. We investigated in a linear mixed effects model whether there was any systematic impact of event repetition on the estimated gain per event, across all participants and event types. This relation was not significant, *F*(19, 173) = 1.00, *p* = .45. We tested the linear term in a separate exploratory model that did not contain any higher‐order polynomial terms for event repetition. In this model, event repetition showed a significantly negative linear relationship with response gain, *F*(1, 191) = 7.07, *p* = .0085. It is therefore possible that habituation in the peripheral (sweat gland) system (but not electrode drift away from the nerve) explains some of the variability in the response gain over trials.

### 3.2 Experiment 2: Intraneural stimulation

**Figure 4:**
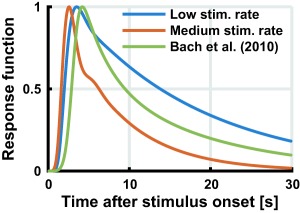
Experiment 2. Estimated response function at low (below 0.15 Hz) and medium (0.19 Hz–0.6 Hz) stimulation rate. Green: Previous SCR model (Bach, Flandin et al., 2010)

**Figure 5:**
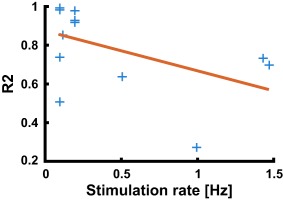
Experiment 2. Coefficient of determination for the regression of SCRF onto previous SCRF model (blue), and linear trend obtained from robust regression (red)

**Figure 6:**
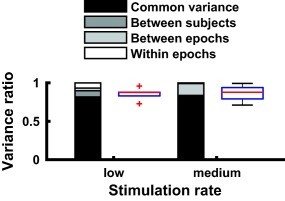
Experiment 2. Ratio of variance in SCR signal, at low (below 0.12 Hz) and medium (0.19 Hz–0.6 Hz) stimulation rate epochs. Bar charts show variance components across epochs under the assumption of a variable gain factor. Black: Common variance, explained by a canonical SCRF across all subjects. Dark gray: Between‐subjects variance, additionally explained when using one SCRF per subject. Light gray: Between‐epochs variance, additionally explained when using one SCRF per epoch. White: Within‐epoch variance, unexplained by the LTI models. Box plots: Variance explained with one SCRF per epoch, for each epoch

SCR for each epoch were fitted with a LTI system that takes a series of Gaussian‐shaped SN bursts as input (parameters in Table 2). Epochs with stimulation rate below 0.6 Hz could be fitted in a meaningful way, and the estimated SCRF was consistent with previous findings and with Experiment 1 (Figure 4). At higher stimulation rates, the SCR looked qualitatively different: high SCR in the first few seconds of the epoch, and much lower activity later on. This meant that the SCRF fitted the first SCR but not later ones with lower amplitude and rather different shape. Consequently, the estimated SCRF was qualitatively different from the ones obtained at slower stimulation frequencies. This may indicate nonlinearities in the peripheral system. We then quantified this discrepancy. For each epoch, we computed a regression of the estimated SCRF onto our previous SCRF model (Bach, Flandin et al., 2010), and report the shared variance *R^2^* (Figure 5). At lower frequencies, the estimated SCRF was similar to the previous model (*R^2^* ± *SD*, 84.11 ± 19.13% for low SR < .12 Hz, 6 epochs; 86.60 ± 15.41% for medium SR, 0.19 < SR < 0.6 Hz, 4 epochs). At higher frequencies the estimated SCRF was less similar to the previous model (56.71 ± 25.64%, 3 epochs). We therefore restricted all further analyses to epochs below 0.6 Hz stimulation rate. Next, we addressed how much variance in the SCR data could be explained under an LTI model, fitting one SCRF per epoch. The explained variance in the signal was 93.15% or 98.94%, at stimulation rates of < 0.12 Hz and 0.19–0.6 Hz, respectively. When fitting epochs of similar stimulation rates together in common models, the explained variance was somewhat lower (for low or medium stimulation frequency, 81.77% or 82.93%; Figure 6). Participant‐wise models for epochs with similar stimulation rates yielded explained variance somewhat higher than in the group‐level model (89.76% or 83.57%; Figure 6). When assuming a constant response gain across all stimulations, explained variance was drastically smaller (19.5% or 1.8% for a canonical model).

**Table 2:** Model parameters from Experiment 2

Parameter^a^	Low stimulation rate	Medium stimulation rate
ϑ_1_	2.3051	2.2433
ϑ_2_	2.5653	4.2898
ϑ_3_	0.1517	0.5584
ϑ_4_	−0.0058	−0.1775

a ϑ1– ϑ3 determine the shape of the SCRF. ϑ4 determines the delay with respect to an eliciting SN burst and depends on the precise location of the intraneural stimulation electrode; hence, it has no generalizable interpretation.

We investigated in a linear mixed model whether there was any systematic effect of stimulation number on the estimated response gain per stimulation, across all participants and stimulation rates. In this experiment, both electrode drift away from the sudomotor fibers, or saturation of the sweat gland system, could lead to a negative relationship with stimulation repetition. We used all epochs with stimulation frequency below 0.6 Hz for this analysis. This relation was not significant, *F*(299, 629) = 0.76, *p* = .99. In particular, a negative linear term in this relation may indicate habituation. We tested for this in a separate exploratory model that did not contain any higher‐order polynomial terms for stimulation repetition. In this model, stimulation repetition showed a significantly positive linear relationship with response gain, *F*(1, 894) = 14.77, *p* < .001, AIC = 1679.45. We also modeled the linear effect of time, rather than of stimulation repetition—this is not the same because the stimulation frequency differed between epochs. In this analysis, we confirmed a linear relationship of time with response gain, *F*(1, 894) = 10.01, *p* = .002, AIC = 1683.41. However, the AIC difference demonstrates that the model with stimulation repetition fit the data significantly better than the model with time. Importantly, neither model indicated an influence of electrode drift nor sweat gland saturation, as the linear relationship was positive (i.e., higher amplitude gain later in the epoch).

## 4 DISCUSSION

Model‐based SCR analysis rests on assumptions about the effector organ system that describe how sudomotor action potential bursts generate measured SCR via acetylcholine release from nerve terminals, transmitter diffusion, and processes in the sweat glands. All existing methods base this system on the assumptions of linearity and time invariance, but these have not yet been formally quantified using invasive methods. Here, we capitalize on intraneural recordings, and intraneural stimulation under regional anesthesia, to conduct such a formal test of the LTI model. Three key findings emerge. In the SN stimulation Experiment 2, we find strong evidence for the LTI model at low rate of SN burst succession. When stimulation frequency is below 0.6 Hz (i.e., fewer than one burst every 1.7 s), around 95% of the variance in the SCR signal can be explained under LTI assumptions. Furthermore, more than 80% can be explained by just one canonical SCRF for all participants, indicating a high degree of similarity between individuals. The estimated SCRF shows good accordance with an SCRF previously developed from sensory stimulation. At the same time, this model breaks down when stimulation frequency exceeds 0.6 Hz. In this case, an LTI model can still explain the data—but the estimated model characteristics (i.e., the SCRF) deviate from those derived at lower stimulation frequencies, or in different experiments. This is in itself a violation of the time invariance principle and indicates nonlinearities in the effector organ system.

As a second finding, the explained variance under LTI assumptions in the intraneural recording Experiment 1 was smaller than in stimulation Experiment 2. On the other hand, a canonical SCRF explained more variance in Experiment 1 than in our previous indirect tests, which additionally addressed variability in the neural system (Bach, Flandin et al., 2010). A possible reason for the higher explained variance in Experiment 2 is that sudomotor and other sympathetic fiber activity could not be separated in Experiment 1, thus contributing to apparent LTI violations. Furthermore, the signal‐to‐noise ratio in the intraneural recordings is by design lower than in the intraneural stimulation. In both experiments, the gain factor (i.e., the proportionality constant between the SN and the SCR signal) was rather variable across trials. In both experiments, exploratory analysis provided weak evidence that the gain factor linearly changed over time, but in a different direction: it decreased in Experiment 1 and increased in Experiment 2. In Experiment 2, stimulation repetition was a better predictor of response gain than time. The linear decrease in Experiment 1 may possibly indicate a saturating sweat gland system, but this was not confirmed in Experiment 2. A larger proportion of variance in response amplitudes was unsystematic. Several possible reasons may account for this. For Experiment 1, in addition to the SN fibers innervating the skin site from which SCR were recorded, SN fibers innervating surrounding skin regions may be corecruited to a variable extent. This would change the amplitude of the SN but not of the recorded SCR signal. In reverse, our SN recordings may have missed fibers innervating the skin region from which we recorded SCR, again leading to variability in the recorded SN amplitude. This is aggravated by the method of determining the skin region innervated by the recorded SN fiber, which was done based on A‐fiber responses to mechanical stimulation—mechanoreception and SN innervation may not entirely overlap. Similarly, in Experiment 2, repeated SN stimulation may have led to activity in different fibers every time, some of which may not innervate the skin region from which SCR were recorded. In summary, these may be reasons why a model fixing response gain explained data in both experiments worse than a model with variable response gain.

Interestingly, the intraneural recording experiment confirmed an earlier assumption—based on nonsystematic inspection of example data—that sudomotor bursts can be approximated by a Gaussian function (Bach, Daunizeau et al., 2010; Bach et al., 2011). The dispersion of this Gaussian was estimated to be 0.3 s in this previous work, which is in the range of our current result.

As a limitation of our current study, the methods of SCR measurement in the two experiments were not identical, and the stimulation frequencies rather different, making a comparison of the obtained SCRF difficult. Also, sample sizes were small, such that generalizability may be limited. Furthermore, because of our peroneal recording method in Experiment 1, SCR may not be fully comparable to the ones obtained at more typical SCR recording sites, for example, at the hand or plantar region of the foot.

We initially argued that three methods—sensory stimulation, microneurography, and direct sudomotor stimulation—can help test the validity of LTI assumptions. We can now identify intraneural stimulation under regional anesthesia as the most sensitive method to do so. Under very controlled experimental conditions, LTI systems can reliably represent the SN/SCR relationship as long as stimulation frequency corresponds to typical psychological or cognitive experiments, that is, well below 1 Hz.

## References

[psyp12986-bib-0001] Alexander, D. M. , Trengove, C. , Johnston, P. , Cooper, T. , August, J. P. , & Gordon, E. (2005). Separating individual skin conductance responses in a short interstimulus‐interval paradigm. Journal of Neuroscience Methods, 146(1), 116–123. https://doi.org/10.1016/j.jneumeth.2005.02.001 15935228 10.1016/j.jneumeth.2005.02.001

[psyp12986-bib-0002] Bach, D. R. (2014). A head‐to‐head comparison of SCRalyze and Ledalab, two model‐based methods for skin conductance analysis. Biological Psychology, 103, 63–68. https://doi.org/10.1016/j.biopsycho.2014.08.006 25148785 10.1016/j.biopsycho.2014.08.006 PMC4266536

[psyp12986-bib-0003] Bach, D. R. , Daunizeau, J. , Friston, K. J. , & Dolan, R. J. (2010). Dynamic causal modelling of anticipatory skin conductance responses. Biological Psychology, 85(1), 163–170. https://doi.org/10.1016/j.biopsycho.2010.06.007 20599582 10.1016/j.biopsycho.2010.06.007 PMC2923733

[psyp12986-bib-0004] Bach, D. R. , Daunizeau, J. , Kuelzow, N. , Friston, K. J. , & Dolan, R. J. (2011). Dynamic causal modeling of spontaneous fluctuations in skin conductance. Psychophysiology, 48(2), 252–257. https://doi.org/10.1111/j.1469-8986.2010.01052.x 20557485 10.1111/j.1469-8986.2010.01052.x PMC3039749

[psyp12986-bib-0005] Bach, D. R. , Flandin, G. , Friston, K. , & Dolan, R. J. (2009). Time‐series analysis for rapid event‐related skin conductance responses. Journal of Neuroscience Methods, 184(2), 224–234. https://doi.org/10.1016/j.jneumeth.2009.08.005 19686778 10.1016/j.jneumeth.2009.08.005 PMC2772899

[psyp12986-bib-0006] Bach, D. R. , Flandin, G. , Friston, K. J. , & Dolan, R. J. (2010). Modelling event‐related skin conductance responses. International Journal of Psychophysiology, 75(3), 349–356. https://doi.org/10.1016/j.ijpsycho.2010.01.005 20093150 10.1016/j.ijpsycho.2010.01.005 PMC2877881

[psyp12986-bib-0007] Bach, D. R. , & Friston, K. J. (2013). Model‐based analysis of skin conductance responses: Towards causal models in psychophysiology. Psychophysiology, 50(1), 15–22. https://doi.org/10.1111/j.1469-8986.2012.01483.x 23094650 10.1111/j.1469-8986.2012.01483.x

[psyp12986-bib-0008] Bach, D. R. , Friston, K. J. , & Dolan, R. J. (2010). Analytic measures for quantification of arousal from spontaneous skin conductance fluctuations. International Journal of Psychophysiology, 76(1), 52–55. https://doi.org/10.1016/j.ijpsycho.2010.01.011 20144665 10.1016/j.ijpsycho.2010.01.011 PMC2877802

[psyp12986-bib-0009] Bach, D. R. , Friston, K. J. , & Dolan, R. J. (2013). An improved algorithm for model‐based analysis of evoked skin conductance responses. Biological Psychology, 94(3), 490–497. https://doi.org/10.1016/j.biopsycho.2013.09.010 24063955 10.1016/j.biopsycho.2013.09.010 PMC3853620

[psyp12986-bib-0010] Bach, D. R. , Gerster, S. , Tzovara, A. , & Castegnetti, G. (2016). A linear model for event‐related respiration responses. Journal of Neuroscience Methods, 270, 147–155. https://doi.org/10.1016/j.jneumeth.2016.06.001 27268156 10.1016/j.jneumeth.2016.06.001 PMC4994768

[psyp12986-bib-0011] Bach, D. R. , & Staib, M. (2015). A matching pursuit algorithm for inferring tonic sympathetic arousal from spontaneous skin conductance fluctuations. Psychophysiology, 52(8), 1106–1112. https://doi.org/10.1111/psyp.12434 25930177 10.1111/psyp.12434 PMC4832284

[psyp12986-bib-0012] Benedek, M. , & Kaernbach, C. (2010a). A continuous measure of phasic electrodermal activity. Journal of Neuroscience Methods, 190(1), 80–91. https://doi.org/10.1016/j.jneumeth.2010.04.028 20451556 10.1016/j.jneumeth.2010.04.028 PMC2892750

[psyp12986-bib-0013] Benedek, M. , & Kaernbach, C. (2010b). Decomposition of skin conductance data by means of nonnegative deconvolution. Psychophysiology, 47(4), 647–658. https://doi.org/10.1111/j.1469-8986.2009.00972.x 20230512 10.1111/j.1469-8986.2009.00972.x PMC2904901

[psyp12986-bib-0014] Bini, G. , Hagbarth, K. E. , Hynninen, P. , & Wallin, B. G. (1980). Thermoregulatory and rhythm‐generating mechanisms governing the sudomotor and vasoconstrictor outflow in human cutaneous nerves. Journal of Physiology, 306(1), 537–552. https://doi.org/10.1113/jphysiol.1980.sp013413 7463376 10.1113/jphysiol.1980.sp013413 PMC1283022

[psyp12986-bib-0015] Boucsein, W. (2012). Electrodermal activity. New York, NY: Springer.

[psyp12986-bib-0016] Boucsein, W. , Fowles, D. C. , Grimnes, S. , Ben‐Shakhar, G. , Roth, W. T. , Dawson, M. E. , & Filion, D. L. (2012). Publication recommendations for electrodermal measurements. Psychophysiology, 49(8), 1017–1034. https://doi.org/10.1111/j.1469-8986.2012.01384.x 22680988 10.1111/j.1469-8986.2012.01384.x

[psyp12986-bib-0017] Buxton, R. B. , Wong, E. C. , & Frank, L. R. (1998). Dynamics of blood flow and oxygenation changes during brain activation: The balloon model. Magnetic Resonance in Medicine, 39(6), 855–864. https://doi.org/10.1002/mrm.1910390602 9621908 10.1002/mrm.1910390602

[psyp12986-bib-0018] Castegnetti, G. , Tzovara, A. , Staib, M. , Gerster, S. , & Bach, D. R. (2016). Assessing fear learning via conditioned respiratory amplitude responses. Psychophysiology, 54(2), 215–223. https://www.ncbi.nlm.nih.gov/pubmed/26950648 27933608 10.1111/psyp.12778 PMC6001548

[psyp12986-bib-0019] Daunizeau, J. , Adam, V. , & Rigoux, L. (2014). VBA: A probabilistic treatment of nonlinear models for neurobiological and behavioural data. Plos Computational Biology, 10(1), e1003441. https://doi.org/10.1371/journal.pcbi.1003441 10.1371/journal.pcbi.1003441 PMC3900378 24465198

[psyp12986-bib-0020] Greco, A. , Valenza, G. , Lanata, A. , Scilingo, E. , & Citi, L. (2015). cvxEDA: A convex optimization approach to electrodermal activity processing. IEEE Transactions on Biomedical Engineering, 63(4), 797–804. https://doi.org/10.1109/TBME.2015.2474131 26336110 10.1109/TBME.2015.2474131

[psyp12986-bib-0021] Hygge, S. , & Hugdahl, K. (1985). Skin conductance recordings and the NaCl concentration of the electrolyte. Psychophysiology, 22(3), 365–367. https://doi.org/10.1111/j.1469-8986.1985.tb01616.x 4011809 10.1111/j.1469-8986.1985.tb01616.x

[psyp12986-bib-0022] Khemka, S. , Tzovara, A. , Gerster, S. , Quednow, B. B. , & Bach, D. R. (2017). Modeling startle eyeblink electromyogram to assess fear learning. Psychophysiology, 54(2), 204–214. https://doi.org/10.1111/psyp.12775 27753123 10.1111/psyp.12775 PMC5298047

[psyp12986-bib-0023] Kirno, K. , Kunimoto, M. , Lundin, S. , Elam, M. , & Wallin, B. G. (1991). Can galvanic skin response be used as a quantitative estimate of sympathetic nerve activity in regional anesthesia? Anesthesia and Analgesia, 73(2), 138–142. https://doi.org/10.1213/00000539-199108000-00006 1854028

[psyp12986-bib-0024] Korn, C. W. , & Bach, D. R. (2016). A solid frame for the window on cognition: Modeling event‐related pupil responses. Journal of Vision, 16(3), 28 https://doi.org/10.1167/16.3.28 10.1167/16.3.28 PMC4993241 26894512

[psyp12986-bib-0025] Korn, C. W. , Staib, M. , Tzovara, A. , Castegnetti, G. , & Bach, D. R. (2017). A pupil size response model to assess fear learning. Psychophysiology, 54(3), 330–343. https://doi.org/10.1111/psyp.12801 27925650 10.1111/psyp.12801 PMC5324687

[psyp12986-bib-0026] Kunimoto, M. , Kirno, K. , Elam, M. , Karlsson, T. , & Wallin, B. G. (1992a). Neuro‐effector characteristics of sweat glands in the human hand activated by irregular stimuli. Acta Physiologica Scandinavica, 146(2), 261–269. https://doi.org/10.1111/j.1748-1716.1992.tb09415.x 1442139 10.1111/j.1748-1716.1992.tb09415.x

[psyp12986-bib-0027] Kunimoto, M. , Kirno, K. , Elam, M. , Karlsson, T. , & Wallin, B. G. (1992b). Non‐linearity of skin resistance response to intraneural electrical stimulation of sudomotor nerves. Acta Physiologica Scandinavica, 146(3), 385–392. https://doi.org/10.1111/j.1748-1716.1992.tb09433.x 1481693 10.1111/j.1748-1716.1992.tb09433.x

[psyp12986-bib-0028] Kunimoto, M. , Kirno, K. , Elam, M. , & Wallin, B. G. (1991). Neuroeffector characteristics of sweat glands in the human hand activated by regular neural stimuli. Journal of Physiology, 442(1), 391–411. https://doi.org/10.1113/jphysiol.1991.sp018799 1798033 10.1113/jphysiol.1991.sp018799 PMC1179895

[psyp12986-bib-0029] Lidberg, L. , & Wallin, B. G. (1981). Sympathetic skin nerve discharges in relation to amplitude of skin resistance responses. Psychophysiology, 18(3), 268–270. https://doi.org/10.1111/j.1469-8986.1981.tb03033.x 7291444 10.1111/j.1469-8986.1981.tb03033.x

[psyp12986-bib-0030] Lim, C. L. , Rennie, C. , Barry, R. J. , Bahramali, H. , Lazzaro, I. , Manor, B. , & Gordon, E. (1997). Decomposing skin conductance into tonic and phasic components. International Journal of Psychophysiology, 25(2), 97–109. https://doi.org/10.1016/S0167-8760(96)00713-1 9101335 10.1016/s0167-8760(96)00713-1

[psyp12986-bib-0031] Macefield, V. G. , Elam, M. , & Wallin, B. G. (2002). Firing properties of single postganglionic sympathetic neurones recorded in awake human subjects. Autonomic Neuroscience, 95(1), 146–159. https://doi.org/10.1016/S1566-0702(01)00389-7 11871781 10.1016/s1566-0702(01)00389-7

[psyp12986-bib-0032] Macefield, V. G. , & Wallin, B. G. (1996). The discharge behaviour of single sympathetic neurones supplying human sweat glands. Journal of the Autonomic Nervous System, 61(3), 277–286. https://doi.org/10.1016/S0165-1838(96)00095-1 8988486 10.1016/s0165-1838(96)00095-1

[psyp12986-bib-0033] Nishiyama, T. , Sugenoya, J. , Matsumoto, T. , Iwase, S. , & Mano, T. (2001). Irregular activation of individual sweat glands in human sole observed by a videomicroscopy. Autonomic Neuroscience, 88(1–2), 117–126. https://doi.org/10.1016/S1566-0702(01)00229-6 11474541 10.1016/S1566-0702(01)00229-6

[psyp12986-bib-0034] Paulus, P. C. , Castegnetti, G. , & Bach, D. R. (2016). Modeling event‐related heart period responses. Psychophysiology, 53(6), 837–846. https://doi.org/10.1111/psyp.12622 26849101 10.1111/psyp.12622 PMC4869677

[psyp12986-bib-0035] Staib, M. , Castegnetti, G. , & Bach, D. R. (2015). Optimising a model‐based approach to inferring fear learning from skin conductance responses. Journal of Neuroscience Methods, 255, 131–138. https://doi.org/10.1016/j.jneumeth.2015.08.009 26291885 10.1016/j.jneumeth.2015.08.009 PMC4612446

[psyp12986-bib-0036] Vallbo, A. B. , Hagbarth, K. E. , Torebjork, H. E. , & Wallin, B. G. (1979). Somatosensory, proprioceptive, and sympathetic activity in human peripheral nerves. Physiological Reviews, 59(4), 919–957. 227005 10.1152/physrev.1979.59.4.919

[psyp12986-bib-0037] Wallin, B. G. , & Elam, M. (1997). Cutaneous sympathetic nerve activity in humans In Gibbins I. L. & Morris J. L. (Eds.), Autonomic innervation of the skin (pp. 111–132). Sydney, Australia: Harwood Academic Publishers.

